# An innovative modelling proposal for the blood supply chain

**DOI:** 10.1111/vox.70234

**Published:** 2026-03-29

**Authors:** Jorge Pagán‐Ortiz, Josué Pagán, María Luisa Lozano, Vicente Vicente, Francisca Ferrer‐Marín

**Affiliations:** ^1^ Medicine Department University of Murcia Murcia Spain; ^2^ Centro Regional de Hemodonación Murcia Spain; ^3^ Electronic Engineering Department Universidad Politécnica de Madrid Madrid Spain; ^4^ Centre for Computational Simulation, Universidad Politécnica de Madrid Madrid Spain; ^5^ Hematology Department Hospital Universitario Morales‐Meseguer, Centro Regional de Hemodonación Murcia Spain; ^6^ CIBERER‐ISCIII CB15/00055 (U765) Murcia Spain; ^7^ Faculty of Medicine Universidad Católica San Antonio (UCAM) Murcia Spain; ^8^ IIS‐Fundación Jimenez Díaz Madrid Spain

**Keywords:** blood donation, blood supply chain, optimization

## Abstract

Whole‐blood donation and apheresis are the only ways to obtain blood components for human use. Tools are being developed to improve the efficiency of blood donation systems by reducing waste, increasing donations and preventing shortages. These tools also help manage the storage and distribution challenges posed by blood products with different expiry dates. The proposed approach focuses on identifying inefficiencies in the ‘blood transfusion chain’ and presents a renewed, practical model that differs from other theoretical ones. It outlines 11 processes across three periods: before donation (promotion), during donation and after blood processing. The goal is to optimize each process, reduce inefficiencies and propose improvements based on current scientific knowledge. While changes to healthcare policies are outside of its scope, the model aims to streamline existing donation processes. This paper emphasizes the need for a ‘vein‐to‐vein’ system, which tracks blood from donor to recipient, managed by a single entity controlling the blood centre's data infrastructure. Though limitations exist due to incomplete control over the transfusion chain, future work will demonstrate the model's application, focusing on areas like scheduling and monitoring pre‐donation haemoglobin levels for better optimization. Comprehensive tracking, including of the recipient, is essential for full system optimization.

**Highlights**

- The study identifies and characterizes 11 interconnected processes of the blood supply chain, covering pre‐donation, donation and post‐processing stages.
- The most frequent inefficiencies—including scheduling, advertising, attempt, collection and quality control—are analysed as key limitations affecting system performance.
- Solutions and optimization strategies reported in the literature are synthesized and structured to support the development of a feasible modelling framework for existing blood donation systems.
- The proposal focuses on optimization in real operational environments, not on redesigning or creating new supply chain structures.

## INTRODUCTION

Blood donation (BD) is currently the only way to obtain human blood, as other production methods are not sufficiently developed to be implemented on an industrial scale [1]. Blood components (BCs) can be obtained either by fractionation of whole‐blood donations, which is the most common method, or by individual component donation through apheresis. Regardless of the donation method, at least three different components (red blood cells, plasma and platelets) are obtained, which must be stored and subsequently distributed.

Most countries lack a standardized approach to calculating regional or national blood needs—although some countries, such as Japan, have studied the problem to predict future needs at the national level [2]. Based on a systematic review of the supply problems and general needs of the BD system, we analyse and delineate the operational boundaries of the blood supply chain (BSC) to identify the main sources of inefficiency and guide the development of an applicable modelling framework. The objective of this research is to establish a basic theoretical framework for the application of modelling, simulation and optimization of a BD centre. Our approach aims to identify inefficiencies within an operating BD centre. Unlike other theoretical industrial models in the literature, this work is a conceptual review and modelling proposal that synthesizes current literature and operational experience to define an optimization framework applicable to real, functioning BD systems, rather than theoretical designs developed from scratch.

## BACKGROUND: GLOBAL PROBLEMS AND LOCAL SOLUTIONS IN THE BSC

Although the problems facing the BD system are global, local vicissitudes and the organization of each administrative system have led to the adoption of completely different solutions. These approaches try to achieve the same general goals: (a) increase donation efficiency and donor participation [3, 4, 5, 6], (b) minimize BC wastage through improved logistics and storage [7, 8, 9] and (c) enhance overall system performance and sustainability [10, 11]. In our framework, donation‐related processes (*ε*_s_ − *ε*_c_) and post‐processing or wastage‐related processes (*ε*_t_ − *ε*_u_) are analysed separately to identify the main optimization opportunities. However, most solutions are applied to specific processes within the BSC. These targeted solutions fail to consider other processes, as well as the relationships and dependencies between them, resulting in a lack of a holistic view of the BSC. As pointed out by Osorio et al. [12], ‘an efficient blood supply chain should meet demand while at the same time reducing wastage and minimizing costs’. This justifies the need to redefine the BSC, regardless of the different configurations it may present in the real world and in the literature.

**FIGURE 1:**
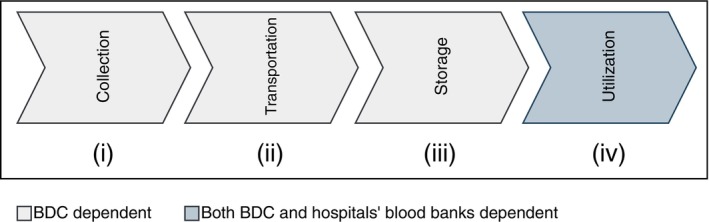
Traditional four‐process representation of the transfusion chain, known as the blood supply chain. BDC, blood donation centre.

Traditionally, the BSC has been depicted as a diagram of four processes: (i) donation, (ii) transportation, (iii) storage and (iv) utilization (Figure 1). However, many authors have addressed specific problems of BD centres that are not reflected in this scheme. This makes the generalization of specific solutions for one BD system and their adaptation to another a complicated task [13].

There is a significant amount of valuable literature on modelling and operational management of the BSC. This literature can be adapted to the needs and processes of the organization [13]. The published knowledge focuses on (i) studies on the optimization of a single transfusion chain process, which includes the development of ideal scenarios; (ii) studies that design and redesign the BSC and describe ideal scenarios that are very complex and potentially useful for developing regions [10, 14]; and (iii) very comprehensive state‐of‐the‐art review articles that explain and collect existing information [10, 11, 15, 16, 17]. Thus, according to the problem they address, we have been able to identify the shortcomings and group the solutions found in the literature into the following key points:

1. Allocation of a BD centre for a new BD system; for the redesign of an existing one, as has been reported for the Turkish Red Crescent blood services [18], or for Colombia [19].
2. Appointment of donors (Donor's recruitment), where voluntary blood donors (remunerated or not remunerated) come to donate blood at a specific place at a specific time [3, 4]. In these cases, the timing of the donation process is critical.
3. Blood collection process [5, 6], where the stages (rooms and medical supplies) and the processes of admission, identification and collection are controlled.
4. Blood bank control and storage [7, 8] to avoid excess or deficit of blood products.
5. Use and transfusion, minimizing the problem of bag waste and unnecessary hospital requests [9]. This describes ‘vein‐to‐vein’ systems that track blood products from donor to recipient, improving patient care by optimizing blood use with up‐to‐date data. However, few BD centres fully integrate such a system because of the need for a single management entity to control the BD‐centre's data infrastructure.

The following section builds upon this review to present our conceptual modelling framework, which integrates these findings into a unified, applicable structure for existing BD systems.

## PROPOSED MODELLING FRAMEWORK AND OPTIMIZATION CRITERIA FOR THE BSC

**FIGURE 2:**
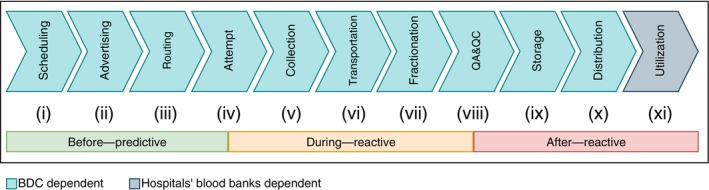
Representation of the suggested 11‐step scheme—divided into three periods: Before attempt (predictive period to make predictions), during donation and after processing (reactive periods for optimization)—of the new blood transfusion supply chain. BDC, blood donation centre; QA&QC, quality assurance and quality control.

To achieve our goal, we outline an operational modelling framework consisting of 11 interconnected processes that can be operated individually or considered holistically. In our methodology, we divide the timeline of the BSC into three periods: processes before donor attempts, also known as ‘Donation Promotion’ (predictive period); processes during donation; and processes after blood processing (both reactive periods). All of the preceding tasks aimed at attracting donors allow us to make predictions (a priori) based on past data, which facilitates the selection of optimal strategies. Once donor attempts occur, predictability decreases and we can only optimize based on the evolving nature of these processes (reactive). Each of the processes in Figure 1 is broken down into 11 sub‐processes, as shown in Figure 2, in a temporal and relational manner. These independent but interrelated modules enable adaptation to centralized or decentralized BD systems.

**FIGURE 3:**
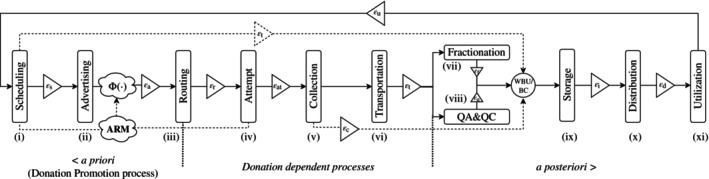
Diagram of the new model of the blood donation process describing efficiency losses. ARM, anaemia remote monitoring; WBU/BC, whole blood unit/blood component; *ε*_a_, advertising error; *ε*_at_, attempt error; *ε*_c_, collection error; *ε*_d_, distribution error; *ε*_i_, inventory error; *ε*_f_, fractionation error; *ε*_q_, quality assurance and quality control error; *ε*_r_, routing error; *ε*_s_, scheduling error; *ε*_t_, transportation error; *ε*_T_, total error; *ε*_u_, utilization error; Φ(·) determines targets.

In Figure 3, a model diagram is presented, where the inefficiencies that lead to the reduction of the BC obtained have been considered. The inefficiencies are represented by the letter ‘*ε*’. The total error *ε*_T_ is measured from the estimation in the ‘Scheduling’ process until the useful BC is obtained. We propose, as a goal, to reduce each of the inefficiencies.

The two predictive processes that we have identified as underpinning the BSC (both constitute what is known as Donation Promotion)—before the donor arrives at the BD centre, ‘a priori’—are (i) scheduling and (ii) advertising. Although documented solutions to scheduling problems exist in the literature, to the best of our knowledge, the processes involved in Donation Promotion (scheduling and advertising) have not previously been included as part of any BD transfusion chain scheme.

**FIGURE 4:**
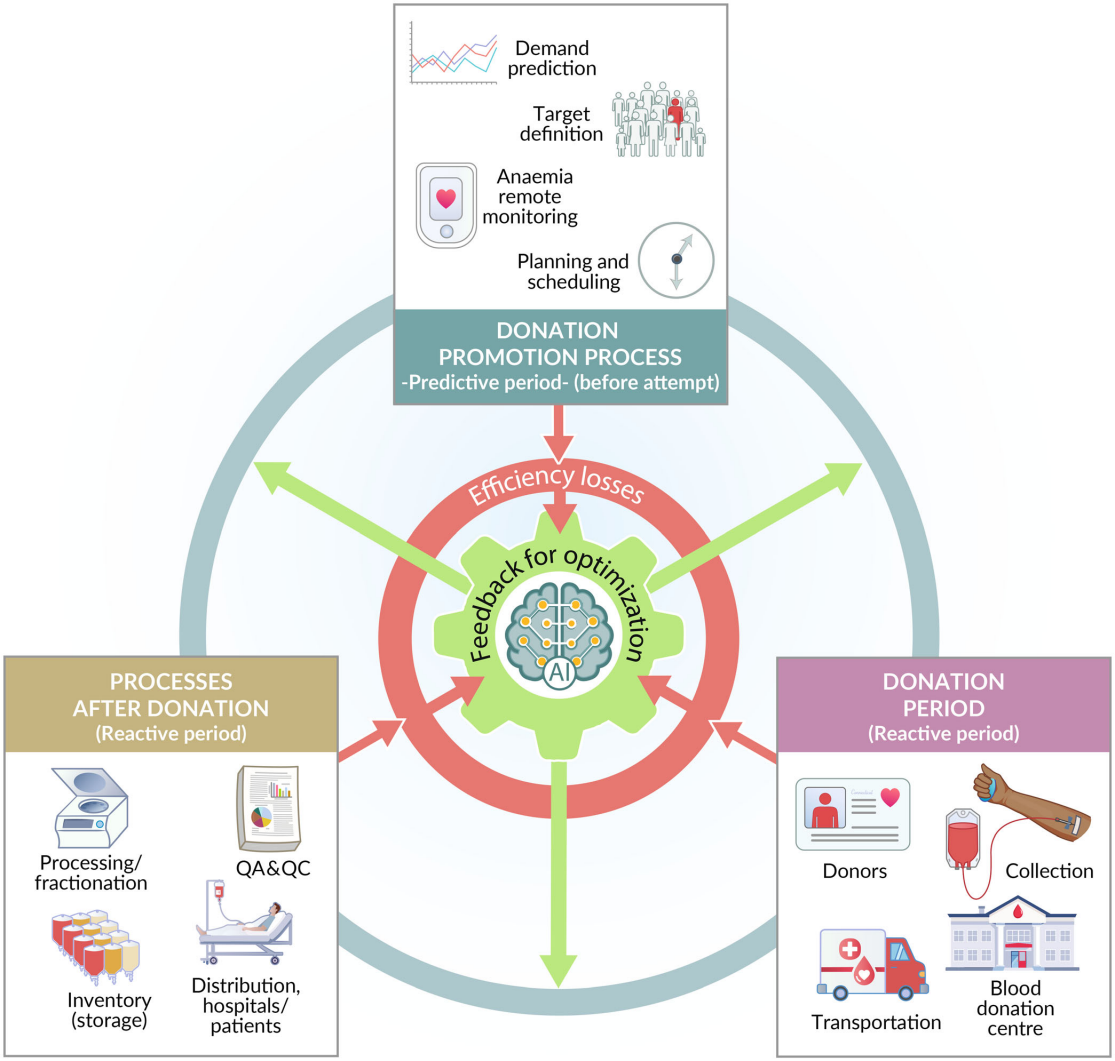
General conceptual description of the proposed modelling framework. Visual summary of the integrated blood supply chain model proposed in this study, illustrating the interaction between predictive and reactive processes, the inefficiencies that ultimately affect the availability of blood components and the importance of continuous feedback for optimizing the entire chain, incorporating proprietary strategies such as the proposed remote monitoring of anaemia. AI, artificial intelligence.

Figure 4 presents a conceptual overview of the proposed modelling framework.

## DESCRIPTION OF THE NEW WORKFLOWS OF THE PROPOSED BSC AND DEFINITION OF INEFFICIENCIES ‘*ε*’

**FIGURE 5:**
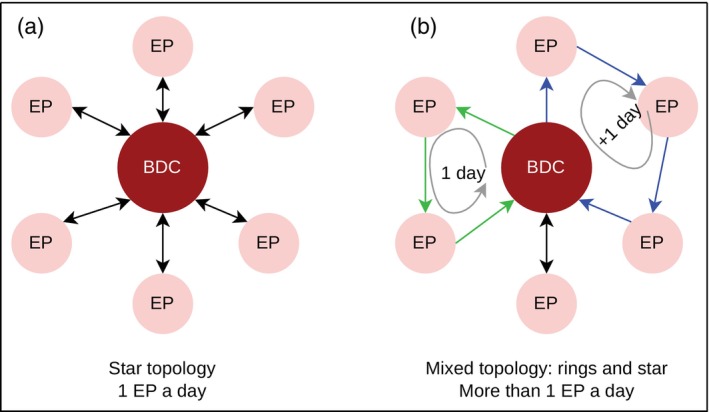
Diagram describing several models of the routing process describes in the literature. (a) Centralized ‘discontinuous’. (b) Centralized ‘continuous’. (b. Green loop) A bloodmobile visits several extraction points (EPs) in a day. (b. Blue loop) A bloodmobile visits several EPs in more than 1 day. BDC, blood donation centre.

1. *The blood collection scheduling process* involves planning collection sites and forecasting the influx of donors. This process begins weeks or months in advance to ensure the availability of venues such as educational centres, businesses or public areas. It uses predictive models to adjust blood requirements based on forecasts of blood bank inventory. Traditional optimization models based on demand history are used in this process [1].
  The scheduling error (*ε*_s_) is the number of people who are expected to participate compared to those who ultimately make an attempt—or in other words, go to the blood collection/extraction points (EPs). Notably, the attempt is not always associated with optimal donation. Inadequate scheduling first affects advertising management. It also affects the effectiveness of the entire BSC.
2. *Advertising*: Advertising effectiveness for donor recruitment varies by the EP type (fixed or mobile centre) and is different for each BD centre and region, and it has evolved over time. It is like a black box; Φ(·) determines goals, but actual participation remains uncertain. In the literature, three major differences in citation have been reported that affect the entire process:
  - Regular donors are appointed by the BD centre with minimal advertising [6], which significantly reduces both *ε*_a_ and *ε*_at_, as shown in Figure 3.
  - Regular donors request an appointment on demand, which requires more scheduling effort and is implemented in advanced BD centres. This channel changes the way appointment optimization is done [20].
  - Regular donors are not scheduled; efforts are focused on mass advertising campaigns. Donors, new or regular, arrive at the EP without appointments or prior notification. This is achieved through various outreach methods such as broadcast, posters, social media or public address systems near the site.
  Advertising error (*ε*_a_) is the ratio of actual donors to the number of potential donors approached.
3. *Routing* involves the movement of personnel and equipment from the BD centre to the EP, known as logistics. Three models can be defined: (i) Centralized ‘discontinuous’: A bloodmobile goes to and returns from an EP (Figure 5a). (ii) Centralized ‘continuous’: A bloodmobile visits several EPs before returning to the BD centre (green loop in Figure 5b). (iii) Shuttle: Visiting several EPs takes more than 1 day (blue loop in Figure 5b) [21].
  Routing error (*ε*_r_) can result from insufficient supplies to meet estimated donations. Several papers address the optimization of routing problems for bloodmobiles [22, 23, 24]. However, some focus only on optimizing tour problems.
4. *Donation attempt* refers to the act of a donor presenting themselves for BD, encompassing both the selection of donors based on guidelines and their consent or willingness to donate, dividing the BSC into pre‐ and post‐attempt phases. Once a donor arrives at the EP, Scheduling and Advertising goals are considered met. Donors undergo a medical interview, including haemoglobin measurements. There are proposals to predict haemoglobin levels [25], and remote monitoring of donors prone to anaemia could optimize visits to prevent rejection. This concept is called anaemia remote monitoring (ARM). By using ARM, we can address potential problems before the donation process, making it more efficient [26].
  The application of donor selection criteria leads to initial rejections, resulting in the loss of potential donations, which is referred to as the attempt error (*ε*_at_). This process is distinct from *Advertising*, which refers to donors who do not attend; *ε*_at_ therefore captures losses that occur only after the donor's arrival. This error is significant both quantitatively, as it represents a loss of whole blood units (WBUs), and qualitatively, as it may discourage donors from returning due to a sense of rejection. Attempt error comes mainly from people who do not donate but should (false‐negative donors, FNDs). Issues in the attempt management include queuing theories and Markovian arrival models, as studied by Alfonso et al. [6] when the population is known. Osorio et al. [19] note that the optimization of both WBUs and apheresis donations together has rarely been studied. They propose a methodology to optimize donor allocation to meet demand while minimizing costs and the number of donors required.
5. *At the Collection*, the donation itself is performed. Collection error (*ε*_c_) (see Equation 1) is the sum of the errors due to the acceptance of false‐positive donors (FPDs) (*ε*_cFP_)—those who proceed to donate and should not—and FNDs (*ε*_cFN_)—those who do not proceed to donate and should.
(1) $$\varepsilon_{c}=\varepsilon_{\mathrm{cFPD}}+\varepsilon_{\mathrm{cFND}}$$
  FPD occurs when a donor is deemed eligible to donate by the interviewer but the donated blood products must later be discarded [27]. Reasons for discarding include problems during donation (e.g., venous access failure or dizziness leading to insufficient blood withdrawal) [28], non‐negative results on disease screening (*ε*_q_) or unacceptable parameters in the BCs (e.g., excess lipids, presence of clots); or post‐donation information (e.g., use of antiplatelet medications that invalidate the use of platelet units).
  FNDs are those who are deferred despite being able to donate and are often subjectively assessed and not always addressed in guidelines due to concerns about donor harm or unexpected adverse reactions. Strategies to reduce deferrals and improve donor retention are critical to avoid blood shortages [29]. As we have previously reported, in our region they are mainly due to misapplication of exclusion criteria or suspicion of unconfirmed anaemia [30, 31], among others. The literature mainly focuses on pre‐donation deferral as FPD [27, 32, 33, 34], either studies evaluating clinical tests or improving pre‐screening processes with community education and awareness [35].
6. *Transportation*: This refers to the process between the EP and the BD centre where ‘Fractionation’ and the remaining steps take place. The WBU should be kept at a temperature of 20–24°C for a maximum of 24 h from collection to fractionation, with monitoring required during transport [36]. Traditional mobile blood collection systems require WBUs to be returned to the BD centre within 24 h of collection [37]. However, new preservation systems integrated into bloodmobiles, known as shuttles, have emerged. These shuttles can temporarily store blood products for more than 24 h, eliminating the need for daily return trips to the BD centre. In regions with more economic resources, these shuttles are typically buses equipped to transport personnel, facilitate donations and transport blood back to the BD centre [21].
  Transportation error (*ε*_t_) is the number of WBUs lost due to exceeded storage time under inappropriate conditions from collection to fractionation.
7. *Fractionation (blood processing)*: During this step, BCs are obtained from the WBUs. This process occurs concurrently with Donation Verification (quality assurance and quality control [QA&QC]). Although fractionation is important for work planning and organization, its importance is relatively low: workloads are typically based on donation forecasts and collection schedules, with occasional adjustments during holiday periods. To manage stock shortages during long weekends or holidays, solutions include increasing collections in the preceding days and using inactivated solutions to extend expiration dates.
  Fractionation error (*ε*_f_) is defined as the rejection of BC due to problems during WBU fractionation [36]. This includes plasma units that contain haemoglobin or appear lipaemic, platelet units discarded due to antiplatelet drug use or low platelet counts and red blood units (RBUs) with haemolysis or clots. In addition, this error category includes units discarded due to broken or damaged contents, as well as cases where donors develop symptoms of illness within 24–48 h of donation, or where inadequate information at the time of donation leads to BC destruction.
8. *QA&QC*: According to Spanish [38] and European legislation [39], all donations must be screened for transfusion transmissible infections (TTIs), including hepatitis B and C virus, HIV, syphilis and other diseases prevalent in specific epidemiological situations. An immunohaematological screening must be performed. In addition, BCs must be subjected to quality control to ensure that they meet established standards [40]. Variability in results is observed between different analytical techniques and blood processing systems, each with its own characteristic degree of variability [41].
  The loss of BC, called quality error (*ε*_q_), is defined as the sum of reactive serological results (both positive and false‐positive [FP] results) [41, 42] and BCs screened for quality control that do not meet established minimum standards. Although this contributes to BC loss, its impact is relatively small.
  The total error (*ε*_T_) (see Equation 2) represents the cumulative loss of usable BCs across all independent processes and is expressed as the sum of individual process errors.
(2) $$\varepsilon_{T}=\varepsilon_{s}+\varepsilon_{a}+\varepsilon_{r}+\varepsilon_{\mathrm{at}}+\varepsilon_{c}+\varepsilon_{t}+\varepsilon_{f}+\varepsilon_{q}$$
  This framework does not aim to establish fixed or ideal error values; instead, it provides a common reference model that enables comparison and process optimization across existing transfusion systems.
9. *Storage (inventory)*: The production of BCs is dependent on consumption and shelf life, which vary from community to community and health system to health system. Although there are no specific guidelines for minimum inventory levels for each BC, a few optimization models address this issue [11, 43]. In addition to accurate forecasting of daily demand and supply, maintaining minimum reserves is critical to avoiding oversupply and wastage of BCs. This forecast is the third most important variable in predictive models for BSC optimization [44].
  Inventory error (*ε*_i_) includes BCs losses due to expiration [45] and losses due to inadequate storage conditions. Traditionally, solutions to these losses rely on FIFO (first in–first out) and LIFO (last in–first out) inventory management systems [14].
10. *Distribution* occurs when a hospital blood bank (HBB) needs BCs and places an order to the BD centre, which then distributes them from its stores, especially in centralized systems. Such requests may be routine (to meet daily hospital needs and maintain baseline inventory), urgent (to meet unexpected demand) or exclusive (or special patient needs). Centralized systems are considered superior to multi‐node systems [11]. Transportation of BCs must comply with specific conditions outlined in Good Manufacturing Practice manuals established by the BD centre [36].
  The error (*ε*_d_) refers to the recall of distributed BCs due to various problems, such as the detection of non‐conformities in BC characteristics or inadequate transportation [46]. While the full impact of this problem remains unclear, the cost of these losses is estimated to be $1.9 million in the United States [46].
11. *Use or transfusion*: This is the final step in optimizing the BSC, as meeting the transfusion needs of BC is the primary goal of the entire process. Improving the BSC includes efforts to refine transfusion indications and maximize the use of BCs [47]. This includes specific training for nurses, technicians and physicians [1]. Notable initiatives in this regard include the European Optimal Blood Use Project [48].
  The margin of use error (*ε*_u_) results from non‐indicated transfusions [49] or from BC reserves for patients who are not transfused (due to overestimation of needs, patient death, etc.).

## OPTIMIZATION OPPORTUNITIES AND ACTIONS TO CONSIDER IN THE WORKFLOWS OF THE PROPOSED NEW BSC

After redefining the BSC and identifying inefficiencies within each process (process errors), Table 1 outlines potential optimization opportunities within these stages. In addition, it lists the necessary considerations that are essential for achieving these improvements. Some optimization opportunities have already been addressed, as we have mentioned in the bibliographic references. However, others still need to be resolved or improved, opening the field to new lines of research for optimizing these points.

**TABLE 1:** Optimization potential and key considerations for improving the inefficiencies (*ε*) described in each link of the new proposed blood transfusion chain.

Process error	To be considered	Optimization opportunity
Pre‐attempt processes (predictive)		
Scheduling error (*ε*_s_): is a forecasting error	Select necessary personnel and establish work groups and schedules; Assess material needs and availability, including mobile units, apheresis equipment and extraction‐donation supplies; Plan the promotion and publicity strategy for the event; Determine the target population and establish donation sites.	Optimization and prediction models can be used based on historical data; Consider seasonal epidemic outbreaks, endemic phenomena and the progression of emerging diseases [50, 51].
Advertising error (*ε*_a_): reflects the impact of donor promotion efforts	Define and specify the target population to be served, which may include employees of private companies, educational institutions or the general public.	Donation promotion strategies should be tailored to each specific population group [52, 53, 54]; Implement pre‐planned mass dissemination campaigns using various channels such as radio broadcasts, poster distribution, mailings, social media campaigns or public address announcements near collection sites.
Routing error (from the BD centre to the EP) (*ε*_r_): supplies to meet planned donation quantities	Topology affects the routing process.	The operating model must be established.
Processes during donation (reactive)		
Attempt error (*ε*_at_): depends on donor characteristics	For walk‐in BD centres, historical statistics can serve as a reference.	Application of an optimization method that minimizes both the cost and the number of donors required; Remote monitoring of anaemia. Predictive and a priori process [26].
Collection (donation) error (*ε*_c_): BD that should or should not have occurred	Well‐defined donors and donations FPD and FN; Assess and measure the impact of human error on the frequency of FND rejections.	Characterize well the profile of the personnel involved in donor selection [55]; Have guidelines with clear selection criteria [56].
Transport error (from EP to BD centre) (*ε*_t_)	‐Distances must be clearly defined and transport conditions must be guaranteed.	‐Determine the losses of WBU due to this cause and improve the routes and storage conditions until fractionation.
Processes after blood processing		
Fractionation error (BC processing) (*ε*_f_)	Know the reasons for product destruction.	Switch to more efficient and safer fractionation systems.
QA&QC error (*ε*_q_): reactive serological results (positive and FPD results)	To know the prevalence of the different infections in the local population [57].	The occurrence of TTI outbreaks should be taken into account; Assess changes and evaluate the impact of introducing new TTI screening techniques [58, 59]; Anticipate changes in WBU extraction kits or BC fractionation and processing techniques that may affect QC results.
Storage error (*ε*_s_): understood as inventory	Each BD centre determines its inventory based on historical needs, storage limitations, donor mobilization capabilities, volume of HBBs served by the BC and other factors These systems achieve greater efficiency when the inventory is viewed holistically, including both the BD centres′ and HBBs' inventories as a single entity. This vein‐to‐vein control is essential for coordinating BC procurement strategies and policies.	To have a system capable of predicting platelet depletion and critical blood groups of the RBU, such as 0Neg.
Distribution error (from BD centre to HBBs) (*ε*_d_)	Reflects human errors such as incomplete orders, shipment of unordered products or loss of orders during distribution. It is essential to know the incidence in each BD centre; Requires a good data communication network.	Robust prediction systems are needed to maintain a more robust BSC, especially in areas where these events occur with high frequency.
Utilization error (transfusion) (*ε*_u_): due to non‐indicated transfusions.	The challenge stems from the difficulty of obtaining the patient data needed to improve the optimization of this process; Reducing transfusion indications can lead to a decrease in BD, affecting the entire transfusion chain [1]; Does not include transfusion‐related product recalls, which are rare and have minimal operational impact [46].	Apply principles of personalized medicine; Adapting the orders of the HBB based on its historical data [60] or predicting the needs of the BC, such as the supply of platelets [44, 61]; Improving the accuracy of estimating blood requirements and fine‐tuning the assessment of patient mortality risk are among the opportunities for improvement.

Abbreviations: BC, blood component; BD, blood donation; BSC, blood supply chain; EP, blood extraction points; FND, false‐negative donor; FPD, false‐positive donor; HBB, hospital blood bank; QA, quality assurance; QC, quality control; RBU, red blood unit; TTI, transfusion transmissible infection; WBU, whole blood unit.

## LIMITATIONS

Although the model proposed does not deal with the clinical use of blood products (patient demand), it must take into account events that occur sporadically and cannot be ignored, such as cases of massive transfusion, although the previous scheme for the availability of these products remains the same.

The model does not include behavioural or motivational determinants of donor willingness, as these are assumed to be reflected in the available donor base. Similarly, clinical blood utilization is treated as a reactive process that informs future scheduling through historical demand, while full vein‐to‐vein traceability remains outside the operational scope of this framework.

The ‘vein‐to‐vein’ supply chain model for blood services is an ambitious undertaking and this document does not attempt to advance the topic by not delving into the transfusion (in‐hospital) part of the chain.

Although only part of this model has been tested with real data and its value evaluated, preliminary results have been presented at conferences on the subject (see references) and will be the subject of future publications.

## CONCLUSION

We have attempted to answer the questions of how to optimize and effectively model the BSC based on the published literature. In addition, we aim to ensure the sustainability and improvement of working models already implemented by providing guidance on how to address areas for improvement within each BSC. This will involve defining and identifying the key points where intervention is required.

The limitations of the system currently in production that have led us to propose a new organization of the BSC are as follows: (i) lack of overall control of the ‘vein‐to‐vein’ paradigm of the transfusion chain; (ii) inability to redesign a system that is in production and that, although not optimized, has proven to be valid; (iii) insufficient ability to implement ‘fine‐grained’ optimizations; and (iv) gradual implementation of the new paradigm of personalized transfusions, which will lead us to be increasingly selective in transfusion indications.

To improve overall performance and achieve more comprehensive management, it is necessary to analyse and calculate losses in each link of the BSC. This requires a holistic review of the entire process and a redefinition of its dependencies and impact on the BSC. At the same time, we have developed a complete optimization model for the entire BSC based on the available scientific literature. By understanding the status of each process, we can generate better plans to manage adverse situations and obtain WBUs. While we recognize that we cannot change health policies, such as introducing paid donations, we can optimize the processes prior to attempting or promoting donations.

With this proposed transfusion chain scheme, we have unified and simplified the links described in other studies while also proposing optimization keys for each link from a realistic and applicable perspective within an established and consolidated BD system. Future work will focus on demonstrating the application of these proposals, especially those based on *Scheduling* and *Advertising*, and the potential for incorporating remotely controllable processes, such as monitoring haemoglobin levels prior to donation.

Finally, the need for vein‐to‐vein monitoring is strongly justified to achieve fully optimized results, including the recipient patient as an integral and optimizable part of the chain.

## CONFLICT OF INTEREST STATEMENT

The authors declare no conflicts of interest.

## Data Availability

All data from this study will be freely accessible upon request to the corresponding authors.
